# Anti-osteoporotic effects of Yi Mai Jian on bone metabolism of ovariectomized rats

**DOI:** 10.3389/fphar.2024.1326415

**Published:** 2024-03-28

**Authors:** Bin Shi, Che-Chun Lin, Chia-Jung Lee, De-Shan Ning, Chao-Chi Lin, Hong-Wei Zhao, Chang-Syun Yang, Shun-Xin Deng, Yung-Jia Chiu, Ching-Chiung Wang

**Affiliations:** ^1^ Infinitus (China) Company Ltd, Guangxhou, Guangdong, China; ^2^ PhD Program for Clinical Drug Development of Herbal Medicine, College of Pharmacy, Taipei Medical University, Guangzhou, Taiwan; ^3^ Graduate Institute of Pharmacognosy, College of Pharmacy, Taipei Medical University, Taipei, Taiwan; ^4^ Traditional Herbal Medicine Research Center, Taipei Medical University Hospital, Taipei, Taiwan; ^5^ School of Pharmacy, College of Pharmacy, Taipei Medical University, Taipei, Taiwan

**Keywords:** anti-osteoporotic effects, Eucommiae Folium, Astragali Radix, Ligustri Lucidi Fructus, Elaeagnus Fructus, Yi Mai Jian herbal formula

## Abstract

Yi Mai Jian herbal formula (YMJ) is formulated with Eucommiae Folium, Astragali Radix, Ligustri Lucidi Fructus, and Elaeagnus Fructus to improve bone function in traditional Chinese medicine. The anti-osteoporotic effects of YMJ in bone metabolism were evaluated in ovariectomized (OVX) rats. The skeletal structure of the femur and vertebrae was analyzed after treating OVX rats with YMJ for 114 days. The results showed that YMJ significantly increased the bone mineral density (BMD) and trabecular number (Tb. N) of the femur and 5th lumbar vertebrae and reduced trabecular separation (Tb. Sp). Moreover, trabecular bone volume/total tissue volume (BV/TV), bone stiffness, and maximum femur load were significantly increased. The serum concentrations of NTX1 and PYD were significantly decreased. According to these results, YMJ could ameliorate osteoporosis in ovariectomized rats. Eucommiae Folium and Elaeagnus Fructus inhibited osteoclast differentiation, Ligustri Lucidi Fructus inhibited calcium reabsorption, Astragali Radix stimulated osteoblast proliferation, and Astragali Radix and Eucommiae Folium stimulated mineralization. Therefore, the combination of the four herbs into one formula, YMJ, could alleviate bone remodeling caused by low estrogen levels. We suggest that YMJ could be a healthy food candidate for preventing post-menopausal osteoporosis.

## 1 Introduction

Osteoporosis, “porous bones,” is a bone disease that causes a reduction in bone mass and density, leading to bone fragility (NIH Consensus Development Panel on Osteoporosis Prevention, Diagnosis, and Therapy, 2001). As human life expectancy increases, osteoporosis has become an important disease of aging, second to cardiovascular diseases. The World Health Organization has recognized osteoporosis as the second most important epidemic worldwide (Cosman et al., 2014). It is estimated that by 2050, hip fractures in Asia due to osteoporosis will reach 3.25 million (Gong et al., 2021). Women are at a higher risk than men, and fractures are more likely to occur in the vertebral body and hip bones. The main reason for this is the major decline on the estrogen levels which consequently increases osteoclast activity after menopausal period. Aging decreases osteoblast growth, resulting to bone degradation that is significantly greater than remodeling. This results in bone loss, decreased bone density, and bone remodeling system imbalance, which, when becomes severe, becomes osteoporosis (Cooper et al., 1992; Li and Wang, 2018). However, there are no ideal preventive and therapeutic drugs for osteoporosis. Therefore, we used the traditional Chinese medicine theory specifically tonic Chinese herbal medicines to develop a new herbal formula, Yi Mai Jian (YMJ), to treat and prevent osteoporosis.

According to the traditional Chinese medicine theory, aging induces weak “qi,” blood, “yin,” and “yang” and causes various physiological dysfunctions. Therefore, we chose the tonic herbs Eucommiae Folium, Astragali Radix, Ligustri Lucidi Fructus, and Elaeagnus Fructus to formulate a therapeutic medicine, YMJ, to improve bone-building functions. Eucommiae Folium, as a “sovereign” medicine in this formula, is a tonifying “yang” herb that can nourish the liver and kidney and strengthen muscles and bones (Lee et al., 2018). At the same time, Astragali Radix is a “minister” medicine that nourishes the “qi” and can secure the exterior and regenerate muscle (Nozaki et al., 2020). Moreover, Ligustri Lucidi Fructus is an “assistant” medicine that can be paired with Astragali Radix to promote bone growth; it also helps in replenishing “yin,” strengthen the muscles and bones, improves vision, and hair blackening (Xiao et al., 2018). In addition to augmenting the protective effect of bones, it also ameliorates dimming eyes and blurring of vision. Elaeagnus Fructus, a “courier” medicine with a sour and slightly sweet taste, promotes digestion (Amiri Tehranizadeh et al., 2016); when included in YMJ, it can reconcile the taste between herbs so that the effect can be mitigated and suitable for the elderly.

Hormones, cytokines, and drugs can cause osteoporosis (Rachner et al., 2011). Estrogen decline plays a vital role in the bone remodeling system. It is commonly used to establish an animal model of post-menopausal osteoporosis (Yousefzadeh et al., 2020). Estrogen reduces bone cell apoptosis, slow down bone remodeling, and inhibit oxidative stress, NF-κB activity, and osteoblast apoptosis, thus continuing bone formation. RANKL binds to osteoclast receptors and promotes their differentiation, leading to an increased rate of bone resorption. Estrogen protects bones through these mechanisms (Soysa and Alles, 2019). Thus, removal of ovaries causes drop in serum estrogen secretion. After more than 90 days post-ovariectomy, rats may develop osteoporosis; an animal model that was used in the present study. The ovariectomized rats were assessed for trabecular bone volume/total tissue volume (BV/TV), trabecular thickness (Tb. Th), trabecular number (Tb. N), trabecular separation (Tb. Sp), bone mineral density (BMD), and bone stiffness. The serum was analyzed as a biomarker of bone turnover. The anti-osteoporotic effects of YMJ were evaluated by analyzing the obtained data (Liu et al., 2015; Kuo and Chen, 2017). RANKL-induced osteoclast differentiation of RAW 264.7 cells were used as a target to evaluate the cellular mechanism of YMJ and its components in bone remodeling (Kim et al., 2019).

## 2 Materials and methods

### 2.1 Chemicals and reagents

Triton X-100, silver nitrate, naphthol AS-MX phosphate disodium salt, dexamethasone, glycerophosphate, ascorbic acid, 17α-Ethynyl estradiol, and 3-(4, 5-Dimethylthiazol-2-yl)-2,5-diphenyltetrazolium bromide (MTT) were purchased from Sigma-Aldrich (St. Louis, MO, United States). Isoflurane was obtained from Aesica Queenborough Limited (Kent, UK). Minimal essential medium alpha (MEM-α) was supplied by Gibco BRL (Grand Island, NY, United States). RANKL was obtained from PeproTech Inc. (Rocky Hill, NJ, United States). 10% formaldehyde solution was purchased from Macron Fine Chemicals (PA, United States).

### 2.2 Preparation of Yi Mai Jian and the extract of its constituent herbs

YMJ formula extract was obtained from Infinitus (China) Company Ltd. (Guangzhou, China) and included Eucommiae Folium (the dried leaf of *Eucommia ulmoides* Oliv.), Astragali Radix (the dried root of *Astragalus membranaceus* (Fisch.) Bunge), Ligustri Lucidi Fructus (the dried ripe fruit of *Ligustrum lucidum* W.T.Aiton (Fam. Oleaceae)), and Elaeagnus Fructus (the dried ripe fruit of *Elaeagnus angustifolia* L.) extracts. The four herbs were purchased from a traditional Chinese medicine store in Taipei and were verified by Lih-Geeng Chen (National Chiayi University, Chiayi, Taiwan). Voucher specimens were deposited at the Graduate Institute of Pharmacognosy, College of Pharmacy, Taipei Medical University (Taipei, Taiwan). The four herbs were sliced and mixed together as a teabag. It was boiled using distilled water with 20 times the weight of herbal medicine for 30 min. The extracted solutions were filtered and evaporated on a rotary evaporator. The herbal extracts were then lyophilized into a powder. The powder was labeled EuF (Eucommiae Folium), AsR (Astragali Radix), LLF (Ligustri Lucidi Fructus), and ElF (Elaeagnus Fructus), and stored at 4 °C.

### 2.3 Ovariectomy-induced osteoporosis rat model

A total of 48 female Sprague Dawley rats (12 weeks of age) weighing 250 ± 10 g were purchased from BioLASCO Taiwan (Yilan, Taiwan). Animals were taken care of in accordance with the Ethical Regulations on Animal Research of Taipei Medical University (approval No.: LAC-2018-0552). Rats in the sham (n = 8) and ovariectomy groups (OVX, n = 40) were housed in standard rat cages on a 12-h light/dark cycle and provided with a standard pellet diet and water *ad libitum*. Ovariectomy was performed via bilateral ovariectomy, and the sham group underwent a sham surgery where rats were anesthetized using isoflurane (Chen et al., 2015). Two weeks post-surgery, the rats were randomly divided into five groups as follows: 1) control group (treated with distilled water); 2) positive control (PC) group (treated with 0.05 mg/kg 17α-Ethynyl estradiol); 3) YMJ groups (treated with 0.31, 0.93, and 1.55 g/kg of YMJ). The sham group was treated with distilled water. The animals’ weight measurements were taken daily, and the volume of YMJ dissolved in distilled water was administered orally according to body weight for 114 days. Rats were sacrificed after the treatment. Serum was collected to determine the biochemistry, and the femur and 5th lumbar vertebrae bones were harvested to determine the structure and material of bone tissue.

#### 2.3.1 Micro-computed tomographic (micro-CT) analysis

The microarchitecture of the femur and 5th lumbar vertebrae was determined using a SkyScan 1,076 Micro-CT (Bruker Belgium S.A./N.V., Kontich, Belgium) at a nominal resolution of 18 μm/pixel to reconstruct the femur and 5^th^ lumbar vertebrae bone (Chen et al., 2015). The specific area of interest (ROI) was 150 consecutive slices of the upper femur metaphysis from the growth plates to the proximal metaphysis, except for cortical bone. Bone mass density was analyzed in the same area, including the cortical bone. A 3D image was generated using the CTAn software (Bruker Belgium S.A./N.V.). Bone morphometric parameters included trabecular bone volume/total tissue volume (BV/TV), trabecular thickness (Tb. Th), trabecular number (Tb. N), trabecular separation (Tb. Sp), and bone mineral density (BMD).

#### 2.3.2 Biomechanics assessment

A modified three-point bending method was used in the current study as previously described (Chen et al., 2019), using a bone and wound breaking strength tester (Model TK-252C; Muromachi Kikai, Tokyo, Japan). The distance between the two loading points was 16 mm, and a press force at a rate of 0.5 mm/min was applied to the midpoint of the femur bone until the bone broke. The biomechanical parameters measured were maximum load (N), energy (mJ), and stiffness (N/mm).

#### 2.3.3 Serum biochemical markers of bone turnover analysis

Blood was collected by cardiac puncture and centrifuged at 3,000 rpm for 15 min to collect serum, and stored at −80 °C. Serum levels of the cross-linked N-telopeptide of type I collagen (NTX1), osteocalcin (OC/BGP), and pyridinoline (PYD) were assayed using a Rat NTX1 ELISA Kit, Rat OC/BGP ELISA Kit, and Rat-PYD ELISA Kit (Elabscience, Houston, TX, United States), respectively. The assays were performed in accordance with the manufacturer’s protocol contained in the kit. The optical density was determined using a spectrophotometer set at 450 nm.

### 2.4 Analysis of osteoclast and osteoblast activity

#### 2.4.1 The cytotoxicity assay of RAW 264.7 cells by MTT assay

The RAW 264.7 macrophage cell line (BCRC 60001) was purchased from the Bioresource Collection and Research Center (Hsinchu, Taiwan). RAW 264.7 cells were seeded in a 96-well plate at a density of 8 × 10^4^ cells/well. The cells were cultured in DMEM media with the test samples for 24 h before MTT assays (Liu et al., 2018). Absorbance was measured at 570 nm using an ELISA reader.

#### 2.4.2 RANKL induced RAW 264.7 cell differentiation to osteoclast cells

RAW 264.7 cells were seeded into a 24-well plate at a density of 2 × 10^4^ cells/well with MEM-α culture media for 2 h before stimulation. Cells were treated with both RANKL (50 ng/mL) and extracts for 4 days. The MEM-α media, RANKL, and extracts were replaced every 2 days (Zhang et al., 2017).

##### 2.4.2.1 Tartrate-resistant acidic phosphatase (TRAP) staining

After culturing, the media was removed, and the plates were washed twice with phosphate-buffered saline (PBS), and cells were fixed with 10% formaldehyde solution for 10 min at 37 °C. The formaldehyde solution was then removed and lysed with 0.1% Triton X-100. After 15 min, the cells were washed twice with PBS and stained with TRAP staining solution for 10 min at 37 °C in the dark. Positive TRAP cells were visualized using a light microscope, and cells containing three or more nuclei were counted as osteoclasts.

##### 2.4.2.2 Pit formation assay

RAW 264.7 cells were seeded into Osteo Assay surface plates (Corning Inc., Corning, NY, United States) (Kim et al., 2019). The differentiation procedure was the same as mentioned above. On the 4^th^ day, the conditioned medium was removed, and the cells were treated with 5% sodium hypochlorite for 5 min. The plates were washed thrice with distilled water, and 5% (w/v) aqueous silver nitrate was added to each well for 30 min in the dark. The plates were removed from the silver nitrate solution and soaked in distilled water for 5 min. The water was discarded, and 5% sodium carbonate was added for 4 min. The sodium carbonate solution was removed, and the plates were dried at 50 °C for 1 h. Five random regions of each well were photographed using a TissueGnostics Axio Observer Z1 microscope (TissueGnostics GmbH, Vienna, Austria), and the resorption area of the chosen regions was counted using HistoQuest (TissueGnostics).

$$Pit\;area\;\left(\%\right)=pit\;area/well\;area\times 100.$$

#### 2.4.3 The proliferation and mineralization of MC3T3-E1 pro-osteoblast assay

##### 2.4.3.1 The proliferation effects of MC3T3-E1 cells by MTT assay

MC3T3-E1 cells (ATCC: CRL-2593, an osteoblast-like cell line from C57BL/6 mouse calvaria) were obtained from the American Type Culture Collection (Rockville, MD, United States). MC3T3-E1 cells were seeded in a 96-well plate at a density of 4 × 10^4^ cells/well. The cells were cultured in MEM-α media with the test samples for 24 h before MTT assays (Liu et al., 2018). Absorbance was measured at 570 nm using an ELISA reader.

##### 2.4.3.2 The analysis of alkaline phosphatase (ALP) activity of MC3T3-E1 cells

MC3T3-E1 cells were seeded in 24-well plates at a density of 7 × 10^4^ cells/well with MEM-α and 10% FBS in a humidified 5% CO_2_ atmosphere at 37 °C for 3 days. To initiate differentiation, 500 μL MEM-α medium containing dexamethasone (10^–7^ M), β-glycerophosphate (10 mM), and ascorbic acid (50 μg/mL) were added to each well. The MC3T3-E1 cells were treated with the test samples during differentiation. The cells were cultured for 5 days, stained with BCIP-NBT solution for 30 min, and photographs were taken using a light microscope.

##### 2.4.3.3 The mineralization effects of MC3T3-E1 cells by alizarin red S staining

To analyze the mineralization effects of MC3T3-E1 cells, the same cultured method was used as for alkaline phosphatase (ALP) activity of the cells. For quantification of mineralization, cells were cultured for 21 days, and the media were replaced every 2 days. Calcium deposition was determined by staining with 0.1% alizarin red S (ARS) for 5 min, and photographs were obtained using a light microscope. To quantify matrix mineralization, cells were destained with 10% cetylpyridinium chloride, and the supernatant was collected to measure the absorbance at 540 nm using an ELISA reader (Liu et al., 2019).

### 2.5 Western blot analysis of osteoclast and osteoblast protein expression

RAW 264.7 cells were induced with RANKL for 3 days, and MC3T3-E1 cells were cultured with differentiation medium for 5 days in preparation for Western blot analysis. Proteins were lysed with RIPA buffer and loaded into SDS-PAGE gels for separation. The gels were transferred to a PVDF membrane (Bio-Rad, Hercules, CA, United States) and blocked with BSA buffer for 1 h. The membranes were incubated with primary antibodies at the specified dilutions: Anti-RUNX2 antibody (1:1000, iReal Biotechnology), DLX2 antibody (1:1000, GeneTex), c-Fos (1:1000, Santa Cruz Biotechnology), Anti-Cathepsin K antibody (1:1000, Abcam) Anti-NFAT2 antibody (1:1000, Abcam), RANK (1:1000, cell signaling), GAPDH (1:5000, Santa Cruz Biotechnology) and β-actin (1:5000, Santa Cruz Biotechnology) overnight at 4 °C. HRP-conjugated secondary antibodies were used at 1 h at room temperature. Bands were visualized by T-Pro LumiLong Plus Chemiluminescent Substrate Kit (T-Pro Biotechnology, Taiwan) and monitored by an iBright™ FL1500 Imaging System (Invitrogen, United States). The quantitative analysis of bands was done using ImageJ software.

### 2.6 Analysis of osteoclast and osteoblast activity

Data were statistically assessed by one-way analysis of variance (ANOVA), followed by Duncan’s test to compare means. *p < 0.05, **p < 0.01, and ****p < 0.0001 were considered statistically significant. All data were presented as mean ± S.D.

### 2.7 Network pharmacology of YMJ on osteoporosis

This study utilized network pharmacology to predict the effects of YMJ on osteoporosis. The potential components of the constituent herbs of YMJ were identified using the Traditional Chinese Medicine Systems Pharmacology Database and Analysis Platform (https://www.tcmsp-e.com/tcmsp.php), and were filtered based on OB > 30% and DL ≥ 0.18. The targets of the potential components of YMJ were then checked using SwissTargetPrediction (http://www.swisstargetprediction.ch/) to ensure a probability score greater than 0. The targets of osteoporosis were identified using OMIM (https://www.omim.org/) and GeneCards (https://www.genecards.org/). The intersection between osteoporosis and YMJ was determined, followed by protein-protein interaction and KEGG pathway analyses.

## 3 Results

### 3.1 Anti-osteoporotic effects of YMJ on OVX rats

**FIGURE 1:**
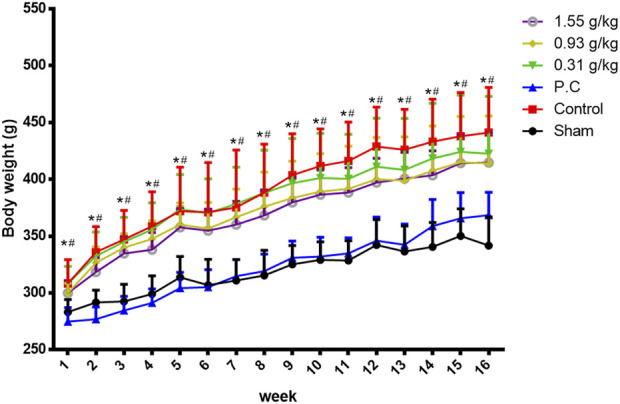
Body weight changed in ovariectomized rats from weeks 1–16. Data are expressed as means ± SD, n = 8. *p < 0.05 sham group compared with the control group. #*p* < 0.05 PC group compared with the control group.

**FIGURE 2:**
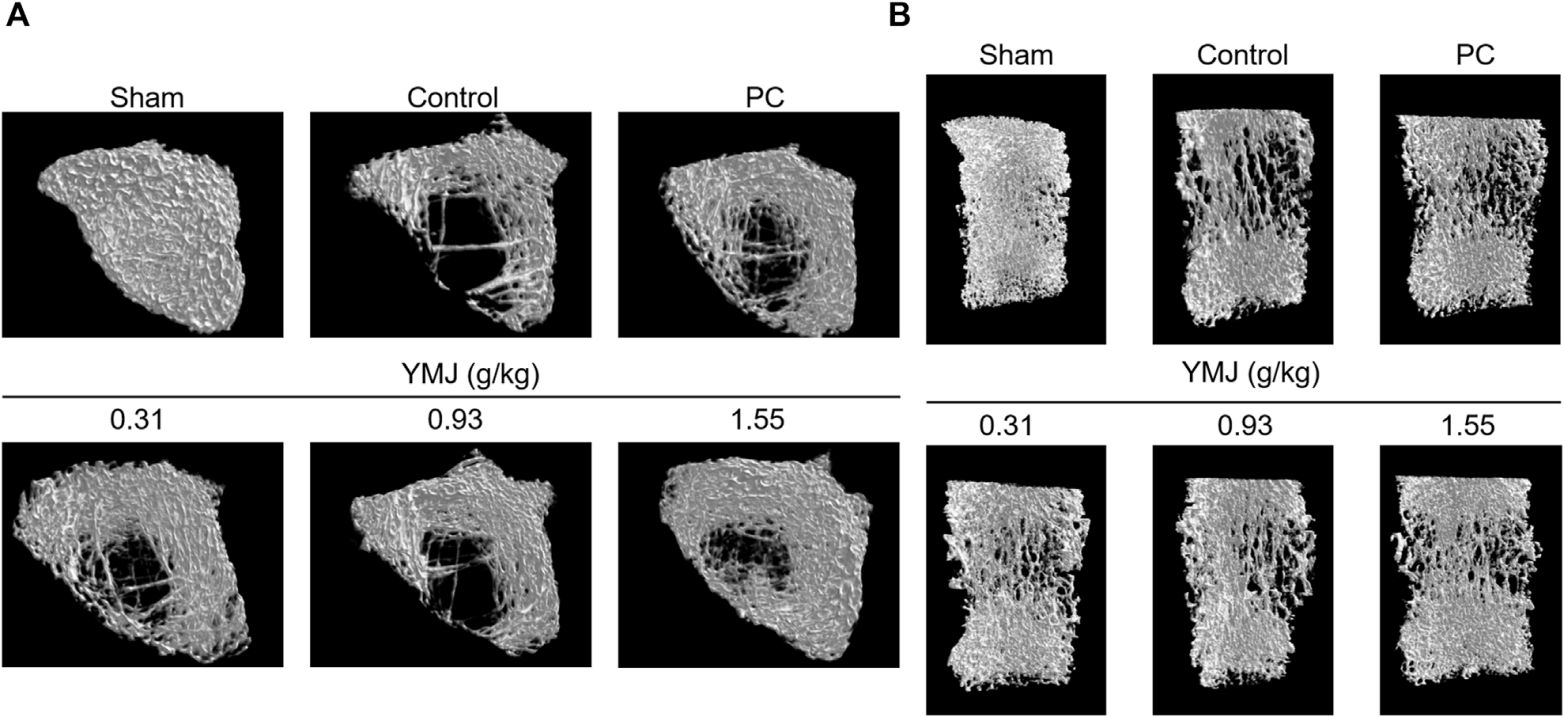
Micro-computed tomography (micro-CT) images of the femur bone **(A)** and the 5th lumbar vertebrae **(B)** after YMJ treatment for 114 days.

Rats were randomly divided into six groups 2 weeks post-surgery. Body weights of the sham and PC groups were significantly lower than those of the control group at 1–16 weeks (Figure 1). The micro-CT microarchitecture image illustrated that YMJ could reduce the trabecular loss of the femur (Figure 2A) and 5^th^ lumbar vertebrae (Figure 2B). The quantitative analysis of micro-CT demonstrated that the BMD in the femur of the 0.31- and 1.55-g/kg YMJ groups was significantly higher than in the control group after 114 days of treatment (Table 1). Moreover, the 1.55-g/kg group also had significantly enhanced BMD in the 5^th^ lumbar vertebrae than the control group (Table 2). Furthermore, BV/TV and Tb. N in the femur of the 0.31- and 1.55 g/kg-YMJ groups were significantly higher, whereas Tb. Sp decreased compared with the control group. However, the 0.31- and 1.55-g/kg YMJ groups only had significantly higher Tb. N in the 5^th^ lumbar vertebrae compared with the control group. There was no significant difference in Tb. Th in the femur and 5^th^ lumbar vertebrae of the YMJ and control groups (Tables 1, 2). Moreover, the biomechanical parameters, stiffness, maximal load, and energy were significantly increased in the 1.55-g/kg YMJ group than in the control group (Table 3).

**TABLE 1:** Bone parameters of the femur from micro-CT at the end of 114 days of YMJ treatment.

Femur	Sham	Control	PC	YMJ (g/kg)		
				0.31	0.93	1.55
BMD	0.81 ± 0.14*	0.19 ± 0.10	0.31 ± 0.16*	0.33 ± 0.09*	0.28 ± 0.07	0.36 ± 0.05*
BV/TV	32.95 ± 4.99*	11.39 ± 3.54	15.12 ± 5.85	16.44 ± 2.99*	14.42 ± 2.45	17.47 ± 1.57*
Tb. N	3.44 ± 0.55*	1.17 ± 0.30	1.73 ± 0.60*	1.68 ± 0.31*	1.47 ± 0.27	1.80 ± 0.17*
Tb. Sp	0.21 ± 0.05*	0.80 ± 0.15	0.47 ± 0.15*	0.56 ± 0.11*	0.69 ± 0.20	0.53 ± 0.10*
Tb. Th	0.10 ± 0.00	0.10 ± 0.01	0.09 ± 0.01*	0.10 ± 0.00	0.10 ± 0.00	0.10 ± 0.01

Data are expressed as mean ± SD, n = 8. *p < 0.05, compared with the control group. BV/TV, bone volume/total tissue volume; Tb. Th, trabecular thickness; Tb. N, trabecular number; Tb. Sp, trabecular separation; BMD, bone mineral density.

**TABLE 2:** Bone parameters of the 5th lumbar vertebrae from micro-CT at the end of 114 days of YMJ treatment.

5th lumbar vertebrae	Sham	Control	PC	YMJ (g/kg)		
				0.31	0.93	1.55
BMD	1.07 ± 0.06*	0.79 ± 0.14	0.80 ± 0.11	0.85 ± 0.24	0.87 ± 0.16	0.94 ± 0.10*
BV/TV	52.61 ± 2.67*	40.19 ± 6.08	40.84 ± 4.66	42.81 ± 10.48	43.84 ± 7.10	47.07 ± 4.18*
Tb. N	4.28 ± 0.29*	2.84 ± 0.41	3.70 ± 0.37*	3.25 ± 0.29*	3.02 ± 0.35	3.53 ± 0.34*
Tb. Sp	0.17 ± 0.01*	0.28 ± 0.06	0.21 ± 0.02*	0.24 ± 0.03*	0.25 ± 0.04*	0.21 ± 0.03*
Tb. Th	0.12 ± 0.01*	0.14 ± 0.02	0.11 ± 0.00*	0.13 ± 0.02	0.15 ± 0.02	0.13 ± 0.02

Data are expressed as mean ± SD, n = 8. *p < 0.05, compared with the control group. BV/TV, bone volume/total tissue volume; Tb. Th, trabecular thickness; Tb. N, trabecular number; Tb. Sp, trabecular separation; BMD, bone mineral density.

**TABLE 3:** Biomechanics assessment of the femur at the end of 114 days of YMJ treatment.

Femur	Sham	Control	PC	YMJ (g/kg)		
				0.31	0.93	1.55
Stiffness (N/mm)	236.5 ± 46.3*	179.4 ± 47.6	236.5 ± 36.9*	213.6 ± 34.0	232.1 ± 20.0*	236.1 ± 43.1*
Maximal load (N)	115.1 ± 25.4	105.3 ± 14.6	115.5 ± 22.3	112.3 ± 13.9	114.9 ± 13.2	128.4 ± 10.3*
Energy (mJ)	37.8 ± 18.2	35.2 ± 11.6	39.5 ± 14.4	41.5 ± 14.1	43.6 ± 11.5	55.7 ± 10.7*

Data are expressed as mean ± SD, n = 8. *p < 0.05, compared with the control group.

### 3.2 YMJ decreased NTX1 and PYD levels on OVX rats

**FIGURE 3:**
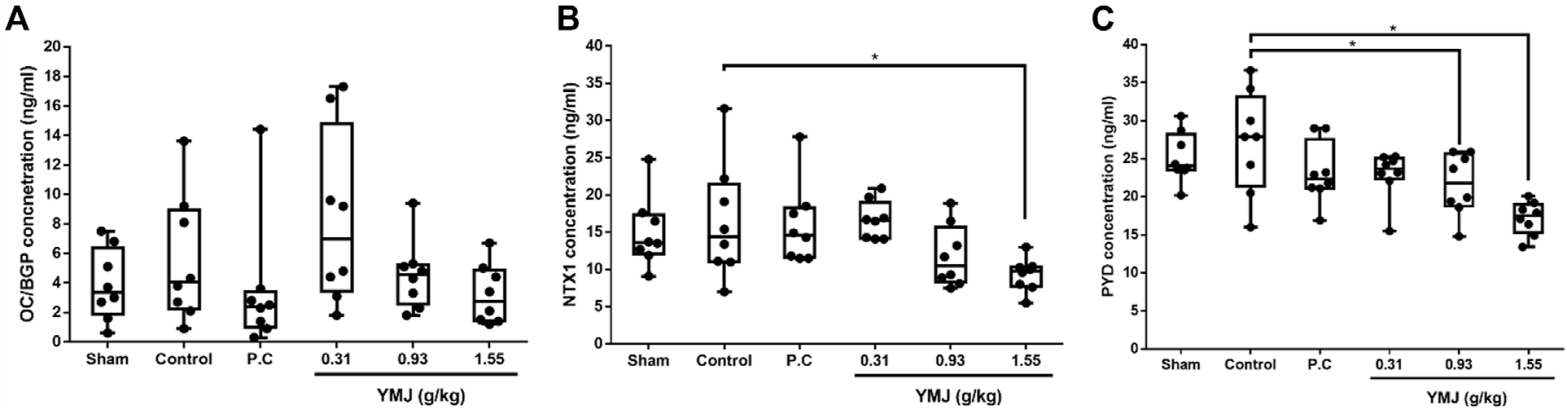
The effect of YMJ on ovariectomized rats on bone turnover biomarkers in serum: OC/BGP (Osteocalcin) **(A)**, NTX1 (Cross-Linked N-Telopeptide of Type I Collage) **(B)**, and PYD (Pyridinoline) **(C)** at the end of the 114-day YMJ treatment. Data are expressed as means ± SD, n = 8. *p < 0.05 compared with the control group.

An increased rate of bone turnover was demonstrated by the generation of serum markers, such as osteocalcin for bone formation and telopeptides of collagen type I and pyridinoline for bone resorption (Hertrampf et al., 2009; Ma et al., 2012; Li et al., 2020). In the current study, there was no significant difference between the sham and control groups. However, YMJ decreased the serum OC/BGP, NTX1, and PYD levels in a dose-dependent manner. In addition, NTX1 and PYD levels were significantly lower in the 1.55-g/kg YMJ group than in the control group (Figure 3).

### 3.3 YMJ and its constituent herbs inhibited osteoclast differentiation and activity

**FIGURE 4:**
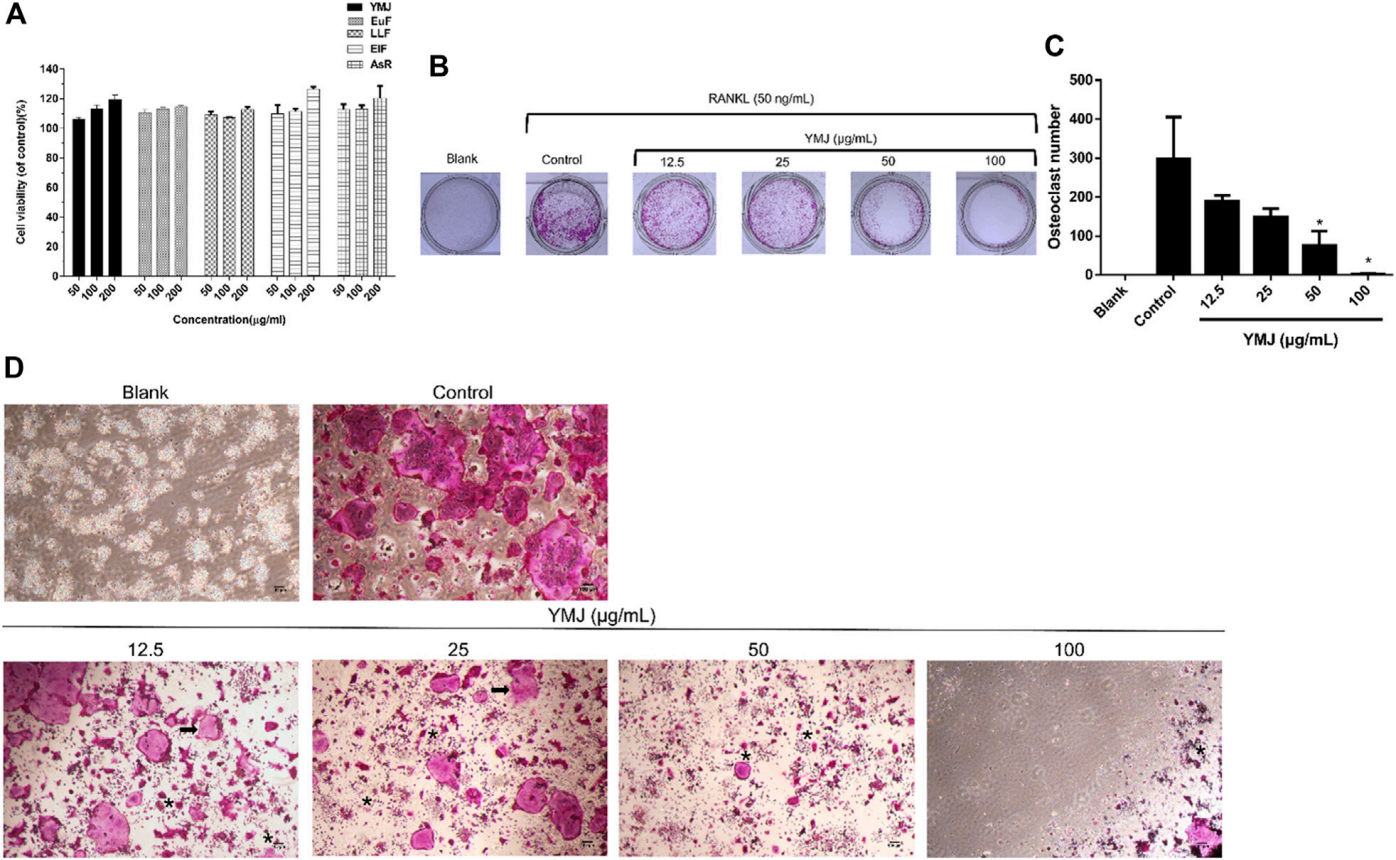
The effects of various concentrations of YMJ on osteoclast cell induction with RANKL (50 ng/mL) were determined using TRAP staining. **(A)** The cell viability of YMJ and the extracts of its constituent herbs in RAW 264.7 cells. **(B)** Representative images were observed in 10-cm dishes. **(C)** TRAP-positive cells were quantified as osteoclasts (nuclei ≥3). *p < 0.05 compared with the control group. **(D)** Representative images were observed using a light microscope (cell death positive programming as indicated by the arrow, apoptotic body as indicated by the asterisks).

**FIGURE 5:**
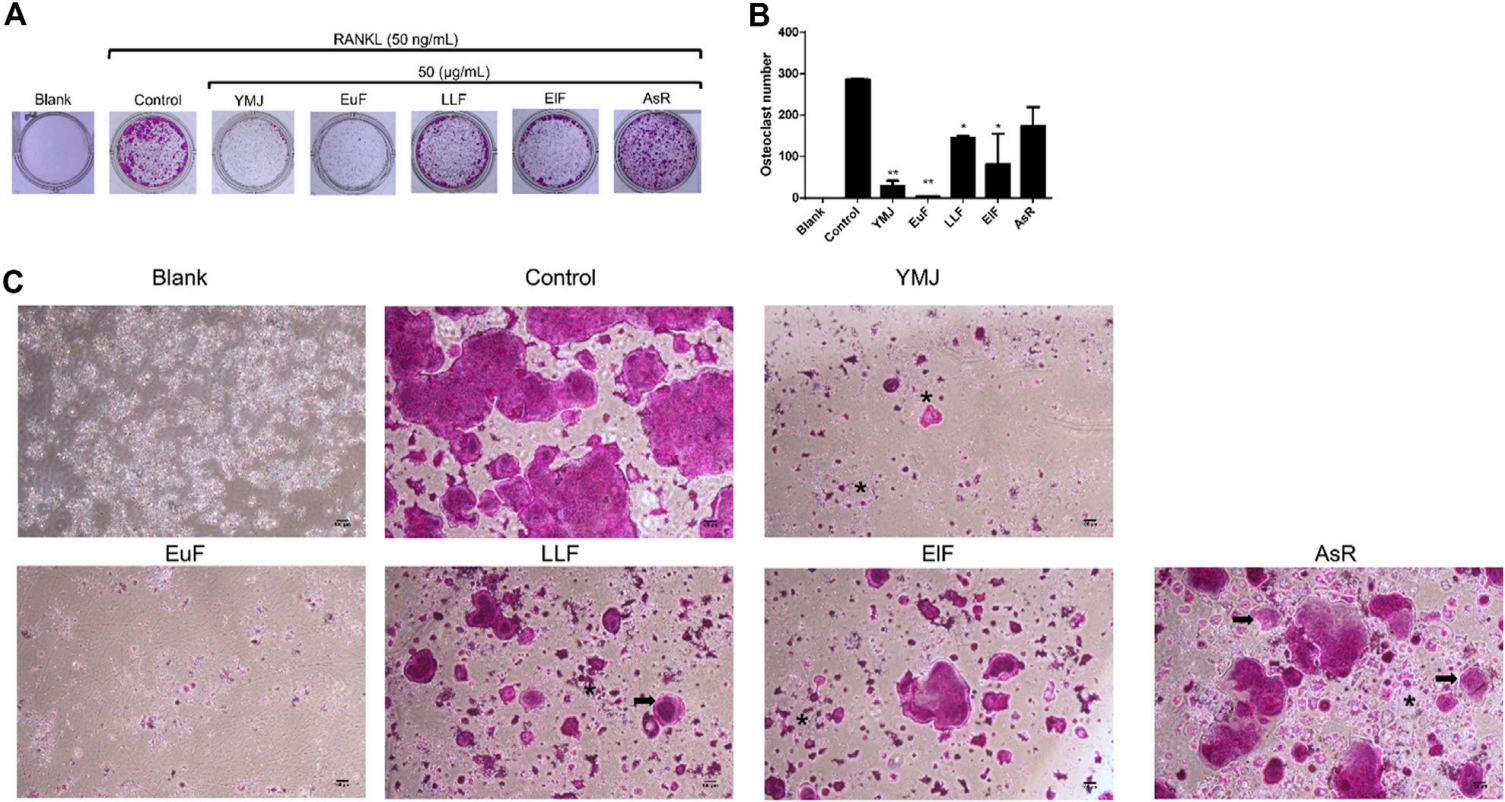
The effects of YMJ and the extracts of its constituent herbs on osteoclast cell induction with RANKL (50 ng/mL) were determined using TRAP staining. **(A)** Representative images were observed in 10-cm dishes. **(B)** TRAP-positive cells were quantified as osteoclasts (nuclei ≥3). *p < 0.05 compared with the control group. **(C)** Representative images were observed using a light microscope (cell death positive programming as indicated by the arrow, apoptotic body as indicated by the asterisks).

**FIGURE 6:**
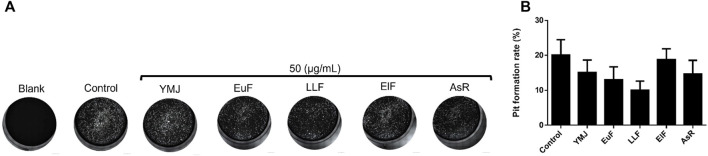
The effects of YMJ and the extracts of its constituent herbs in RANKL (50 ng/mL) induced pit formation areas are shown as images **(A)** and quantified data **(B)** using a TissueGnostics Axio Observer Z1 microscope.

**FIGURE 7:**
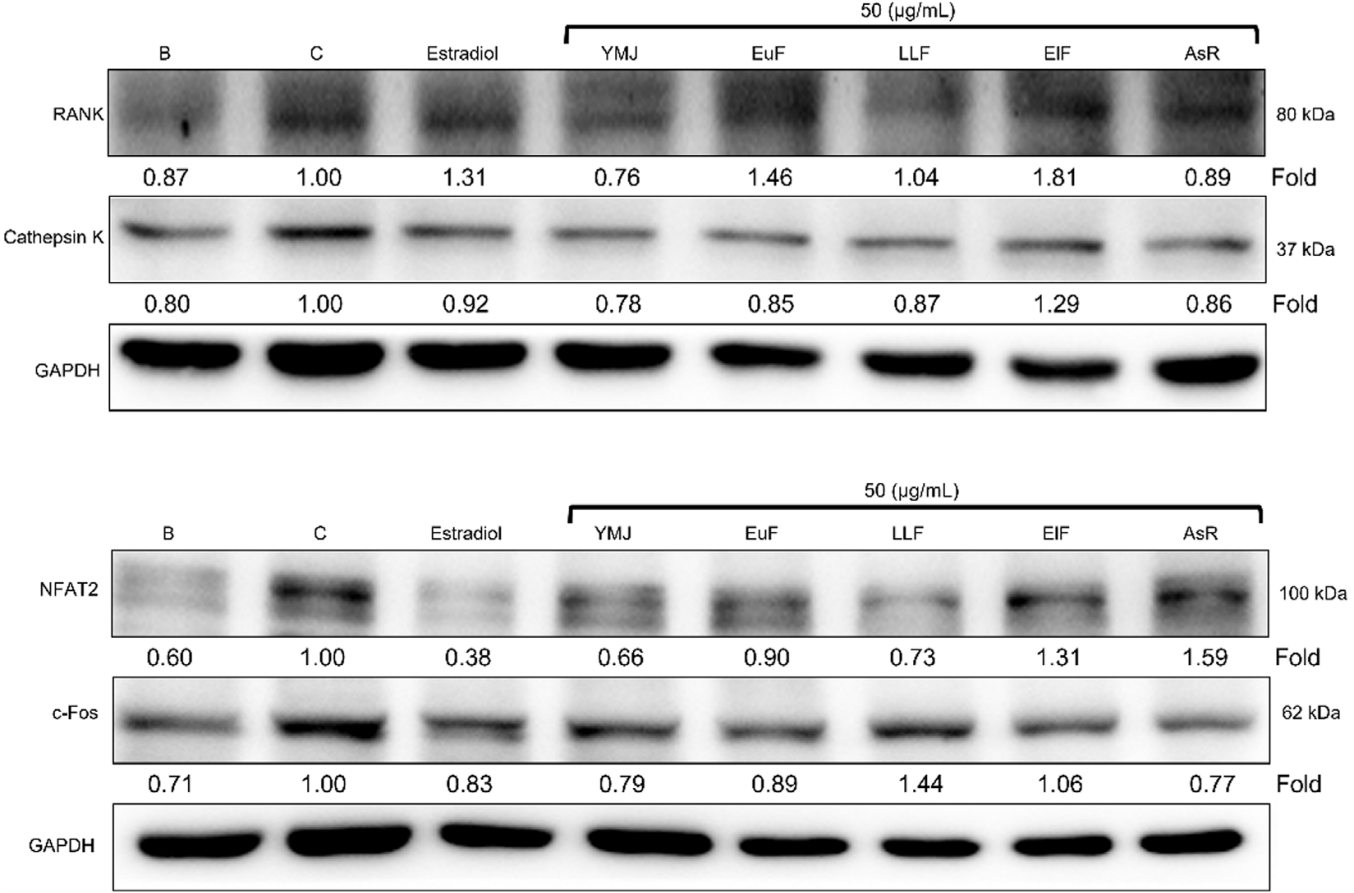
The effects of YMJ and the extracts of its constituent herbs on osteoclast cell induction with RANKL (50 ng/mL) were assessed through protein expression analysis using Western blots. RANK, Cathepsin K, c-FOS, and NFAT2 on osteoclast cell induction with RANKL.

YMJ and its herbs did not show any cell cytotoxic effect on RAW264.7 cells at concentrations below 200 μg/mL (Figure 4A). RANKL induced RAW 264.7 cells to differentiate into osteoclasts, and TRAP staining was used to investigate the effects of YMJ on osteoclast differentiation. Compared with the control group, YMJ had a dose-dependent effect on the inhibition of osteoclast differentiation and significantly reduced the number of TRAP-positive multinucleated osteoclasts (≥3 nuclei) at a concentration of 50 μg/mL or above (Figures 4B–D). Furthermore, at 50 μg/mL of YMJ or its constituent herbs, the inhibition of osteoclast differentiation was in descending order as follows: EuF > YMJ > ElF > LLF > AsR, with no significant differences in the number of TRAP-positive multinucleated osteoclasts, compared with the control group (Figure 5). To investigate the effects on osteoclast function, the cells were assessed using Osteo Assay surface plates. Although there was a significant difference between the control and the other groups, YMJ and the extracts of its constituent herbs still suppressed osteoclastic bone resorption, and the inhibition of osteoclast activity in descending order (LLF > EuF > AsR > ElF > YMJ) (Figure 6). Osteoclast differentiation related gene including RANK, c-FOS, and NFAT2 were upregulated compared to blank in RANKL induced RAW 264.7 cell differentiation for 3 days. Treatment of YMJ reduced RANK, c-FOS, and NFAT2 protein expression relative to control. Similarly, both EuF and LLF inhibited the protein expression of NFAT2 while EuF alone inhibited c-FOS protein expression. Additionally, AsR inhibited RANK and c-FOS protein expression. YMJ, EuF, LLF, and AsR also decelerated the bone resorption capacity of osteoclasts by inhibiting the protein expression of Cathepsin K (Figure 7). The results showed that EuF and ElF were the main constituents reducing osteoclast differentiation, and LLF was the main constituent decreasing osteoclast function.

### 3.4 AsR and EuF stimulated ALP activity and mineralization in MC3T3-E1 cells

**FIGURE 8:**
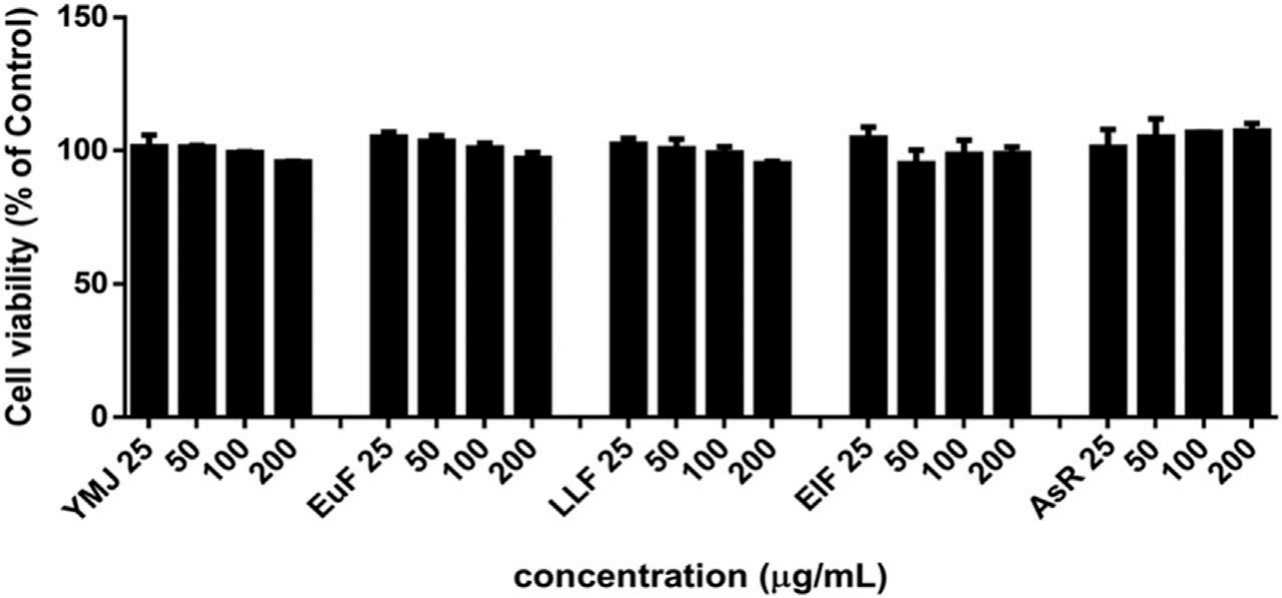
The effects of YMJ and the extracts of its constituent herbs on the proliferation of MC3T3-E1 cells were measured via an MTT assay. MC3T3-E1 cells were treated with EuF, LLF, ElF, AsR, and YMJ (25, 50, 100, or 200 μg/mL) for 24 h.

**FIGURE 9:**
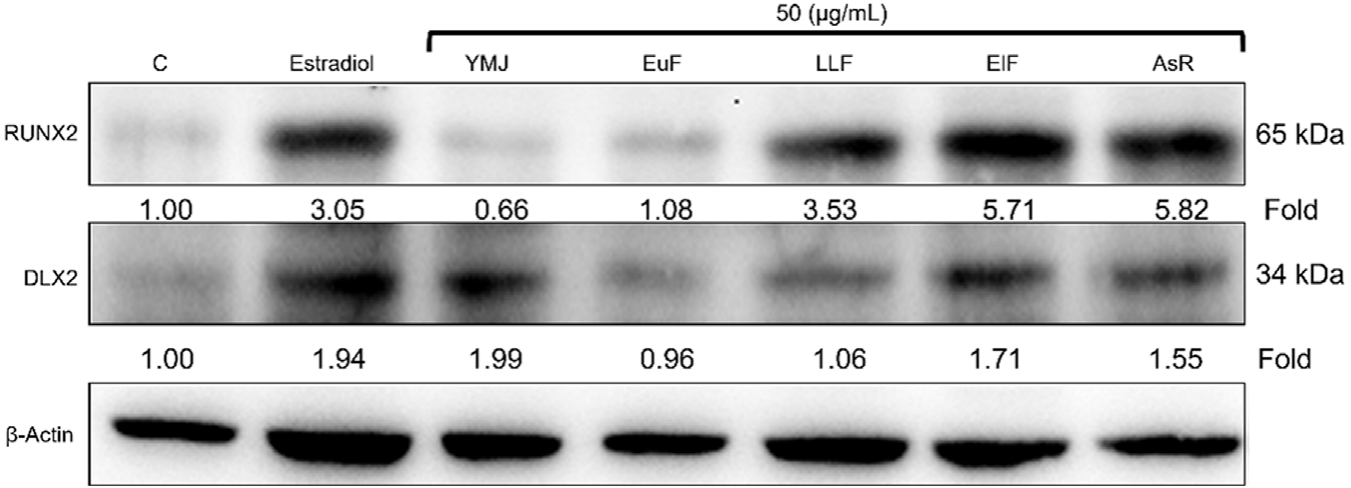
The effects of YMJ and the extracts of its constituent herbs on osteoblast differentiation of MC3T3-E1 cells were assessed through protein expression analysis using Western blots. RUNX2, and DLX2 on osteoblast differentiation of MC3T3-E1 cells.

**FIGURE 10:**
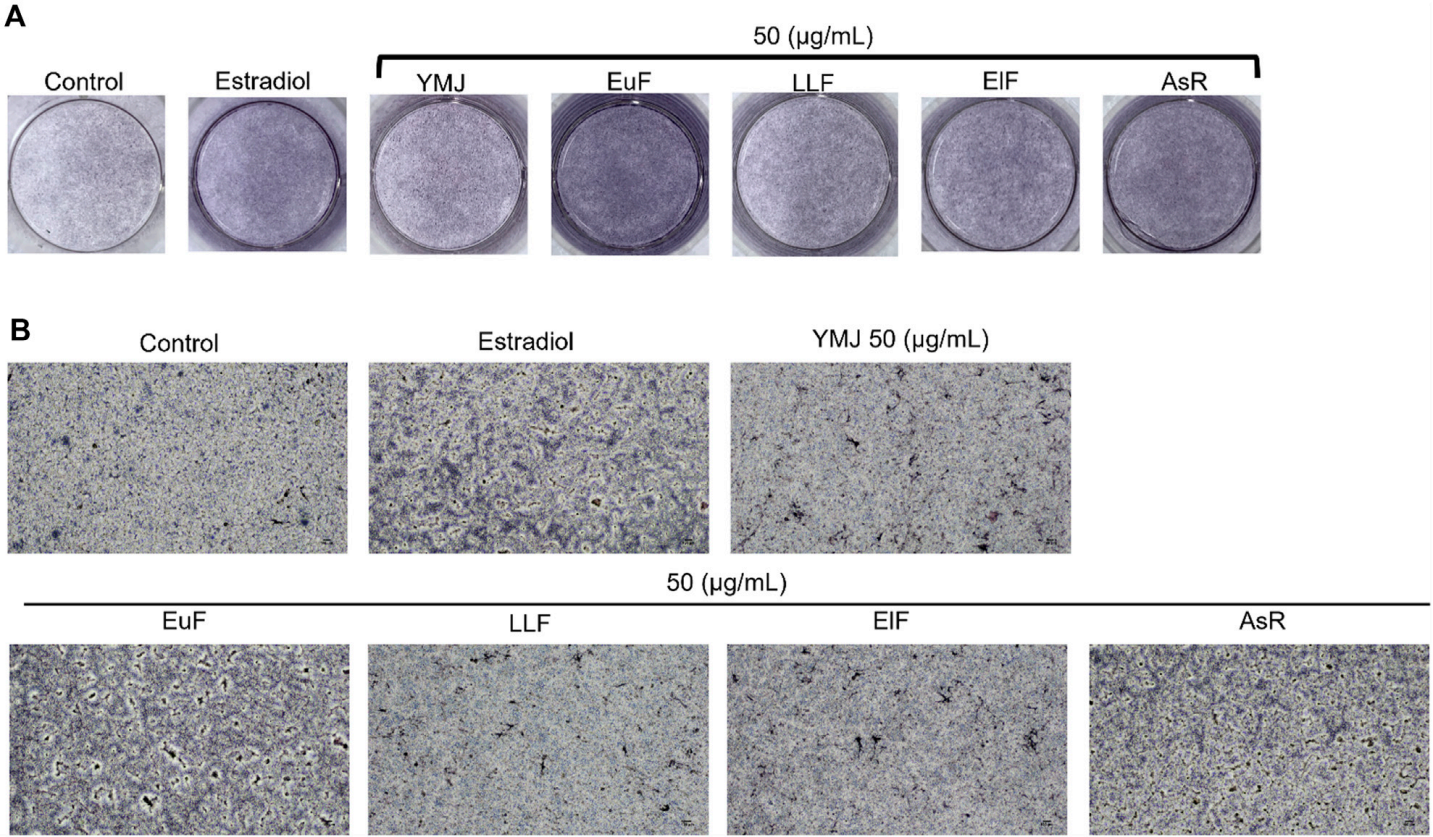
The effects of YMJ and the extracts of its constituent herbs on osteoblast differentiation of MC3T3-E1 cells were determined by ALP activity. Representative images were observed under a culture plate **(A)** using a light microscope **(B)**.

**FIGURE 11:**
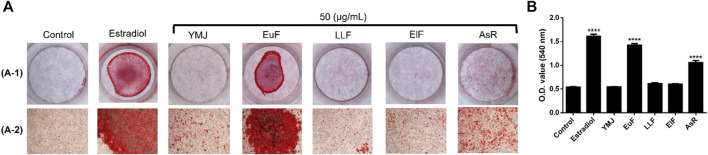
The effects of YMJ and the extracts of its constituent herbs on the mineralization of MC3T3-E1 cells were determined by alizarin red S staining. Representative images were observed under a culture plate (A-1) using a light microscope (A-2). Mineralization was assessed as quantification of alizarin red S staining **(B)**. ****p < 0.0001 compared with the control group. **(A)** included A-1 and A-2.

YMJ and its constituent herbs were non-cytotoxic in MC3T3-E1 cells at concentrations up to 200 μg/mL (Figure 8). Furthermore, AsR increased the proliferation of MC3T3-E1 cells in a dose-dependent manner. The differentiation of osteoblasts was evaluated after 5 days using Western blot analysis and ALP activity, while matrix mineralization was assessed 21 days after ARS staining. The results demonstrated that YMJ and the extracts of its constituent herbs could increase DLX2 protein expression, with ElF having most prominent effect. The constituent herbs increased RUNX2 protein expression in the following order: AsR > ElF > LLF > EuF. EuF and AsR significantly enhanced osteoblast differentiation and mineralization, as evidenced by the appearance of blue and reddish dots as compared to the control group. Also, it was observed that there was a higher correlation between ALP activity and osteoblast mineralization but no significant promotion of osteoblast mineralization was observed between YMJ and control group (Figures 9–11).

### 3.5 Network pharmacology analysis of the effect of anti-osteoporosis of YMJ

**FIGURE 12:**
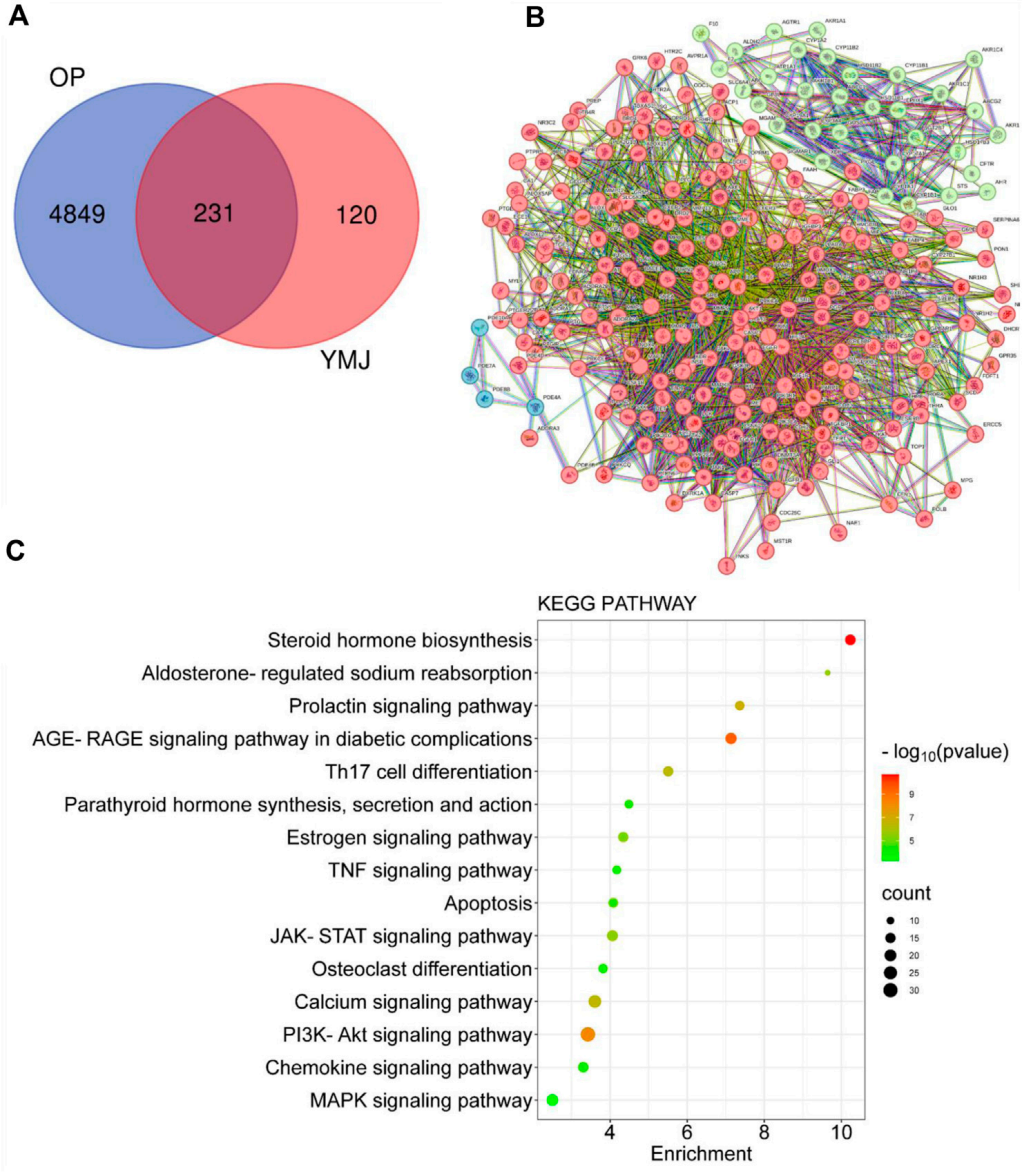
Network analysis of YMJ on osteoporosis. **(A)** Venn diagram targets **(B)** Protein-protein interaction network **(C)** KEGG pathway analysis of YMJ on osteoporosis.

PPI and KEGG pathway analysis were performed on 15 potential components of YMJ containing kaempferol, quercetin, beta-sitosterol, eriodictyol, olitoriside, luteolin, mairin, jaranol, hederagenin, 3S,8S,9S,10R,13R,14S,17R)-10,13-dimethyl-17-[(2R, 5S)-5-propan-2-yloctan-2-yl] 2,3,4,7,8,9,11,12,14,15,16,17-dodecahydro-1H cyclopenta[a]phenanthren-3-ol, isorhamnetin, bifendate, formononetin, isoflavanone, calycosin. The results indicated that YMJ had 231 targets that could influence osteoporosis. It was found to impact hormonal status via the steroid hormone biosynthesis, estrogen signaling pathway, parathyroid hormone synthesis, secretion and action pathways. YMJ may also inhibit osteoclast differentiation through Th17 cell differentiation, TNF signaling pathway, and MAPK signaling pathway. These pathways could be a potential topic in future research to elucidate the mechanistic pathway of osteoporosis amelioration using YMJ, both *in vivo* and *in vitro* (Figure 12).

## 4 Discussion

YMJ comprises Eucommiae Folium, Astragali Radix, Ligustri Lucidi Fructus, and Elaeagnus Fructus. According to the general dosage for traditional Chinese medicine, it recommends an optimal dose of 3 g per administration. Therefore, the estimated required dosage for Wistar rats are 1, 3, and 9 times the human dosage which is approximately 0.31, 0.93, and 1.55 mg/kg, respectively. According to previous studies, the ethanol extract of Eucommiae Folium promotes osteoblast growth through anti-oxidation and activation of ERK and AKT (Zhang et al., 2013; Guan et al., 2021). Hot water extracts can inhibit TRAP activity, which in turn inhibits osteoclast differentiation (Zhao et al., 2020). Astragalus polysaccharides can reduce serum RANKL, osteocalcin, and TNF-α levels in OVX-induced osteoporosis mice and increase the content of osteoprotegerin (OPG) to prevent osteoporosis (Huo and Sun, 2016; Ou et al., 2019). Astragaloside IV promotes osteoblast maturation by enhancing runt-related transcription factor 2 (Runx2), type 1 collagen (COL1A2), and alkaline phosphatase (ALP) (Bian et al., 2011). The ethanol extract of Ligustri Lucidi Fructus has been shown to reduce oxidative stress, improve calcium metabolism, regulate lipid metabolism, and inhibit bone resorption(Chen et al., 2017). The 70% ethanol extract of Elaeagnus Fructus has an inhibitory effect on OVX-induced osteoporosis in rats (Dabbaghmanesh et al., 2017).

We demonstrated that YMJ prevents postmenopausal osteoporosis in an OVX-induced osteoporosis rat model. When osteoblasts are activated, osteocalcin, NTX1, and PYD are produced. Osteocalcin (OC/BGP) and NTX1 were extensively studied as biochemical markers for bone turnover, circulating in the blood and partly excreted via urination. Meanwhile, OC/BGP is a product of osteoblasts, a non-collagenous protein constituting the bone matrix. Peptides like NTX1, ICTP, and CTX-MMP, are generated during bone resorption by osteoclasts degrading collagen. PYD was formed by the further degradation of them and was excreted through urine (Szulc, 2018). In osteoporosis, bone absorption and formation were concurrently promoted, with absorption surpassing formation, leading to bone loss and disrupted metabolic balance in bone tissues. In addition, as bone resorption increases, OC/BGP was released from the bone matrix into the blood, resulting in elevated levels in the blood of osteoporotic individuals. The measurement of serum osteocalcin demonstrates its potential as a diagnostic biomarker for postmenopausal osteoporosis in women (Vakili et al., 2021; Mousavi et al., 2023). In the ovariectomized (OVX) rat model, there was no significant increase in the control group, and it is speculated that the rate of bone remodeling was not significantly different from that of the sham group. However, the serum concentrations of NTX1 and PYD in the experimental group were significantly lower from those in the control group. YMJ treatment also reduced OC/BGP serum levels, demonstrating its beneficial impact on regulating ovariectomy-induced bone turnover and maintaining normal metabolism. It is hypothesized that YMJ could inhibit the activation of osteoclasts and slow down osteoporosis development. In Table 1, the bone parameters of 0.93 g/kg compared with 0.31 g/kg group did not have dose dependent effect. However, there was no significant difference between 0.31 and 0.93 g/kg treated groups. We suggest that the components of YMJ are complicated which can yield a lot of phytochemical substances. The synergistic or antagonistic interactions among various components may lead to non-linear or non-dose-dependent effects.

**FIGURE 13:**
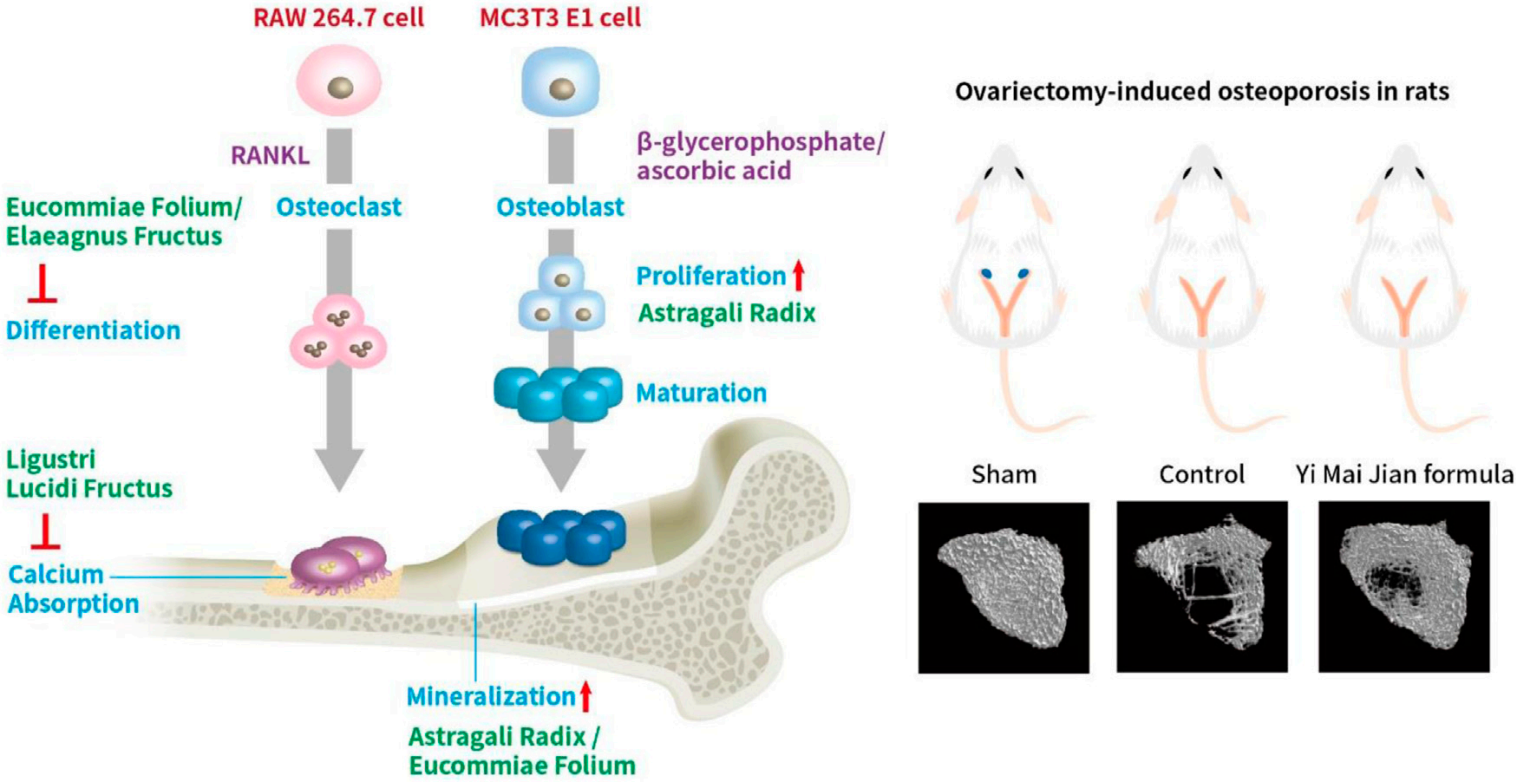
The effects of YMJ and the extracts of its constituent herbs improved osteoporosis through regulating bone metabolism.

The *β*-glycerophosphate-induced osteoblast precursor cell line (MC3T3-E1) maturation test was used to evaluate the effects of YMJ and its components on osteoblast proliferation, maturation, and mineralization. Results indicated that at the same sample concentration, the composition of AsR could significantly stimulate the proliferation of osteoblasts and promote mineralization, while EuF could significantly promote differentiation and mineralization. In the same dosage of YMJ and its constituent herbs, although different herbs had different mechanisms in osteogenesis, YMJ had no significant effect on stimulating osteoblast proliferation and enhancing mineralization. It is inferred that the prescription composition effect did not amount to an additive effect. On the other hand, the differentiation test of the osteoclast precursor cell line (RAW 264.7 cells) induced by RANKL was used to evaluate YMJ and its effect on osteoclast differentiation and calcium absorption activity. Our research results indicated that YMJ and the extracts of its constituent herbs did not cause death in RAW 264.7 cells (Figure 4). The results showed that EuF, LLF, ElF, and YMJ all inhibited osteoclast differentiation. The potency of YMJ’s inhibition of osteoclast differentiation had a compositional additive effect. However, we have observed that YMJ could reduce the number of osteoclasts, possibly by inducing apoptotic bodies (asterisks) and osteoclast death (arrow). Furthermore, our analysis of the KEGG pathway of network pharmacology has also suggested that YMJ could induce an apoptosis pathway (Figure 12). Therefore, we suggested that YMJ inhibited osteoclast formation and induced osteoclast apoptosis (Figure 4). RANKL could bind to the RANK receptor found on the surface of osteoclast precursor cells, which will then trigger the MAPK pathway. This pathway enhanced the expression of c-FOS protein, leading to an increase in the expression of NFAT2 protein. NFAT2 further promoted the expression of osteoclast-related proteins, resulting to osteoclast differentiation (Nakashima and Takayanagi, 2011; Kuroda and Matsuo, 2012). The results indicated that YMJ could inhibit the expression of RANK, c-FOS, and NFAT2 proteins to reduced osteoclasts differentiation. Cathepsin K and MMP break down collagen and minerals which produced bone matrix fragments that were endocytosed by osteoclast cells (Fernandes et al., 2013). Our study demonstrated that YMJ, EuF, LLF, and AsR reduced the protein expression of Cathepsin K (Figure 7). In addition, the ruffled border of osteoclasts plays a crucial role in the process of bone resorption, which is shaped through the extensive fusion of secretory lysosomes (Delaisse et al., 2021; Jurado et al., 2023). Osteoclast cells lacking a ruffled border had no bone resorption capacity. RANKL-induced RAW 264.7 cells with treatment of LLF may result in TRAP positive cells that lacked calcium resorption capability. This may also explain why LLF had a higher inhibition of osteoclast resorption ability. Both YMJ and its herbal constituents could significantly inhibit calcium absorption, and the potency of YMJ is not significantly different from that of the individual constituents, wherein it does not show an additive effect. Based on the results of *in vitro* cell experiments, it is seen that the main YMJ component promoting osteoblast mineralization and inhibiting osteoblast differentiation and calcium absorption is EuF, while AsR had a combination of different effects that can promote osteoblast growth. Both EuF and LLF can inhibit calcium absorption, but the effect is stronger than that of EuF alone, which assists in inhibiting osteoclast activity. ElF is less capable of inhibiting osteoclast differentiation than EuF, but it can induce apoptosis of differentiated osteoclasts and still assist in osteoclast activity inhibition. This research used a rat model with osteoporosis induced by estrogen loss due to OVX. Network Pharmacology found that YMJ may help restore hormonal balance and reduce the effects of osteoporosis. Moreover, an analysis of the KEGG pathway suggested that YMJ had the potential to inhibit osteoclast differentiation by impacting the TNF and MAPK signaling pathways (Figure 12). We successfully produced YMJ based on the traditional Chinese medicine theory which promoted bone growth. Pharmacological experiments indicated that YMJ could prevent osteoporosis. Moreover, the four constituents of YMJ inhibited calcium absorption. In animal experiments, YMJ has been shown to inhibit the production of NTX1 and PYD in osteoclasts. The main mechanism of action is the inhibition of osteoclast activity (Figure 13).

## Data Availability

The original contributions presented in the study are included in the article/Supplementary Material, further inquiries can be directed to the corresponding author.

## References

[B1] Amiri Tehranizadeh Z. Baratian A. Hosseinzadeh H. (2016). Russian olive (Elaeagnus angustifolia) as a herbal healer. Bioimpacts 6 (3), 155–167. 10.15171/bi.2016.22 27853679 PMC5108988

[B2] Bian Q. Huang J. H. Liang Q. Q. Shu B. Hou W. Xu H. (2011). The osteogenetic effect of astragaloside IV with centrifugating pressure on the OCT-1 cells. Pharmazie 66 (1), 63–68. 10.1691/ph.2011.0219 21391437

[B3] Chen B. Wang L. Li L. Zhu R. Liu H. Liu C. (2017). Fructus Ligustri Lucidi in osteoporosis: a review of its pharmacology, phytochemistry, pharmacokinetics and safety. Molecules 22 (9), 1469. 10.3390/molecules22091469 28872612 PMC6151717

[B4] Chen H. L. Tung Y. T. Chuang C. H. Tu M. Y. Tsai T. C. Chang S. Y. (2015). Kefir improves bone mass and microarchitecture in an ovariectomized rat model of postmenopausal osteoporosis. Osteoporos. Int. 26 (2), 589–599. 10.1007/s00198-014-2908-x 25278298

[B5] Chen X. H. Shi Z. G. Lin H. B. Wu F. Zheng F. Wu C. F. (2019). Resveratrol alleviates osteoporosis through improving the osteogenic differentiation of bone marrow mesenchymal stem cells. Eur. Rev. Med. Pharmacol. Sci. 23 (14), 6352–6359. 10.26355/eurrev_201907_18459 31364143

[B6] Cooper C. Campion G. Melton L. J. 3rd (1992). Hip fractures in the elderly: a world-wide projection. Osteoporos. Int. 2 (6), 285–289. 10.1007/BF01623184 1421796

[B7] Cosman F. de Beur S. J. LeBoff M. S. Lewiecki E. M. Tanner B. Randall S. (2014). Clinician's guide to prevention and treatment of osteoporosis. Osteoporos. Int. 25 (10), 2359–2381. 10.1007/s00198-014-2794-2 25182228 PMC4176573

[B8] Dabbaghmanesh M. H. Noorafshan A. Talezadeh P. Tanideh N. Koohpeyma F. Iraji A. (2017). Stereological investigation of the effect of Elaeagnus angustifolia fruit hydroalcoholic extract on osteoporosis in ovariectomized rats. Avicenna J. Phytomed 7 (3), 261–274. 10.22038/AJP.2017.15516.1617 28748173 PMC5511978

[B9] Delaisse J.-M. Søe K. Andersen T. L. Rojek A. M. Marcussen N. (2021). The mechanism switching the osteoclast from short to long duration bone resorption. Front. Cell Dev. Biol. 9, 644503. 10.3389/fcell.2021.644503 33859985 PMC8042231

[B10] Fernandes M. De Ataide I. Wagle R. (2013). Tooth resorption part I-pathogenesis and case series of internal resorption. J. conservative Dent. 16 (1), 4–8. 10.4103/0972-0707.105290 PMC3548344 23349568

[B11] Gong X. F. Li X. P. Zhang L. X. Center J. R. Bliuc D. Shi Y. (2021). Current status and distribution of hip fractures among older adults in China. Osteoporos. Int. 32 (9), 1785–1793. 10.1007/s00198-021-05849-y 33655399

[B12] Guan M. Pan D. Zhang M. Leng X. Yao B. (2021). The aqueous extract of Eucommia leaves promotes proliferation, differentiation, and mineralization of osteoblast-like mc3t3-E1 cells. Evid. Based Complement. Altern. Med. 2021, 3641317. 10.1155/2021/3641317 PMC8238580 34249129

[B13] Hertrampf T. Schleipen B. Offermanns C. Velders M. Laudenbach U. Diel P. (2009). Comparison of the bone protective effects of an isoflavone-rich diet with dietary and subcutaneous administrations of genistein in ovariectomized rats. Toxicol. Lett. 184 (3), 198–203. 10.1016/j.toxlet.2008.11.006 19063953

[B14] Huo J. Sun X. (2016). Effect of Astragalus polysaccharides on ovariectomy-induced osteoporosis in mice. Genet. Mol. Res. 15 (4). 10.4238/gmr15049169 28002602

[B15] Jurado S. Parés A. Peris P. Combalia A. Monegal A. Guañabens N. (2023). Osteoclast generation from RAW 264.7 and PBMC cells. The set up in our lab. Rev. Osteoporosis Metab. Miner. 15 (1), 6–11. 10.20960/revosteoporosmetabminer.00005

[B16] Kim J. H. Kim M. Jung H. S. Sohn Y. (2019). Leonurus sibiricus L. ethanol extract promotes osteoblast differentiation and inhibits osteoclast formation. Int. J. Mol. Med. 44 (3), 913–926. 10.3892/ijmm.2019.4269 31524244 PMC6657961

[B17] Kuo T. R. Chen C. H. (2017). Bone biomarker for the clinical assessment of osteoporosis: recent developments and future perspectives. Biomark. Res. 5, 18. 10.1186/s40364-017-0097-4 28529755 PMC5436437

[B18] Kuroda Y. Matsuo K. (2012). Molecular mechanisms of triggering, amplifying and targeting RANK signaling in osteoclasts. World J. Orthop. 3 (11), 167–174. 10.5312/wjo.v3.i11.167 23330071 PMC3547110

[B19] Lee G. H. Lee H. Y. Choi M. K. Choi A. H. Shin T. S. Chae H. J. (2018). Eucommia ulmoides leaf (EUL) extract enhances NO production in ox-LDL-treated human endothelial cells. Biomed. Pharmacother. 97, 1164–1172. 10.1016/j.biopha.2017.11.035 29136955

[B20] Li D. J. Liu G. Q. Xu X. J. (2020). Silence of lncRNA BCAR4 alleviates the deterioration of osteoporosis. Eur. Rev. Med. Pharmacol. Sci. 24 (11), 5905–5913. 10.26355/eurrev_202006_21483 32572903

[B21] Li L. Wang Z. (2018). Ovarian aging and osteoporosis. Adv. Exp. Med. Biol. 1086, 199–215. 10.1007/978-981-13-1117-8_13 30232761

[B22] Liu L. Wang D. Qin Y. Xu M. Zhou L. Xu W. (2019). Astragalin promotes osteoblastic differentiation in mc3t3-E1 cells and bone formation *in vivo* . Front. Endocrinol. (Lausanne) 10, 228. 10.3389/fendo.2019.00228 31040823 PMC6476984

[B23] Liu M. Fan F. Shi P. Tu M. Yu C. Yu C. (2018). Lactoferrin promotes MC3T3-E1 osteoblast cells proliferation via MAPK signaling pathways. Int. J. Biol. Macromol. 107, 137–143. 10.1016/j.ijbiomac.2017.08.151 28863893

[B24] Liu Z. Yan C. Kang C. Zhang B. Li Y. (2015). Distributional variations in trabecular architecture of the mandibular bone: an *in vivo* micro-CT analysis in rats. PLoS One 10 (1), e0116194. 10.1371/journal.pone.0116194 25625431 PMC4307973

[B25] Ma B. Zhang Q. Wu D. Wang Y. L. Hu Y. Y. Cheng Y. P. (2012). Strontium fructose 1,6-diphosphate prevents bone loss in a rat model of postmenopausal osteoporosis via the OPG/RANKL/RANK pathway. Acta Pharmacol. Sin. 33 (4), 479–489. 10.1038/aps.2011.177 22426695 PMC4003357

[B26] Mousavi S. Vakili S. Zal F. Savardashtaki A. Jafarinia M. Sabetian S. (2023). Quercetin potentiates the anti-osteoporotic effects of alendronate through modulation of autophagy and apoptosis mechanisms in ovariectomy-induced bone loss rat model. Mol. Biol. Rep. 50 (4), 3693–3703. 10.1007/s11033-023-08311-w 36829081

[B27] Nakashima T. Takayanagi H. (2011). New regulation mechanisms of osteoclast differentiation. Ann. N. Y. Acad. Sci. 1240 (1), E13–E18. 10.1111/j.1749-6632.2011.06373.x 22360322

[B28] NIH Consensus Development Panel on Osteoporosis Prevention, Diagnosis, and Therapy (2001). Osteoporosis prevention, diagnosis, and therapy. JAMA 285 (6), 785–795. 10.1001/jama.285.6.785 11176917

[B29] Nozaki R. Hung Y. L. Takagi K. Nakano D. Fujii T. Kawanishi N. (2020). Differential protective effects of Radix astragali, herbal medicine, on immobilization-induced atrophy of slow-twitch and fast-twitch muscles. Biomed. Res. 41 (3), 139–148. 10.2220/biomedres.41.139 32522931

[B30] Ou L. Wei P. Li M. Gao F. (2019). Inhibitory effect of Astragalus polysaccharide on osteoporosis in ovariectomized rats by regulating FoxO3a/Wnt signaling pathway. Acta Cir. Bras. 34 (5), e201900502. 10.1590/s0102-865020190050000002 31166463 PMC6583917

[B31] Rachner T. D. Khosla S. Hofbauer L. C. (2011). Osteoporosis: now and the future. Lancet 377 (9773), 1276–1287. 10.1016/S0140-6736(10)62349-5 21450337 PMC3555696

[B32] Soysa N. S. Alles N. (2019). Positive and negative regulators of osteoclast apoptosis. Bone Rep. 11, 100225. 10.1016/j.bonr.2019.100225 31720316 PMC6838739

[B33] Szulc P. (2018). Bone turnover: biology and assessment tools. Best Pract. Res. Clin. Endocrinol. Metabolism 32 (5), 725–738. 10.1016/j.beem.2018.05.003 30449551

[B34] Vakili S. Zal F. Mostafavi‐pour Z. Savardashtaki A. Koohpeyma F. (2021). Quercetin and vitamin E alleviate ovariectomy‐induced osteoporosis by modulating autophagy and apoptosis in rat bone cells. J. Cell. Physiology 236 (5), 3495–3509. 10.1002/jcp.30087 33030247

[B35] Xiao Y. P. Zeng J. Jiao L. N. Xu X. Y. (2018). Review for treatment effect and signaling pathway regulation of kidney-tonifying traditional Chinese medicine on osteoporosis. Zhongguo Zhong Yao Za Zhi 43 (1), 21–30. 10.19540/j.cnki.cjcmm.20171106.002 29552807

[B36] Yousefzadeh N. Kashfi K. Jeddi S. Ghasemi A. (2020). Ovariectomized rat model of osteoporosis: a practical guide. EXCLI J. 19, 89–107. 10.17179/excli2019-1990 32038119 PMC7003643

[B37] Zhang J. Xu H. Han Z. Chen P. Yu Q. Lei Y. (2017). Pulsed electromagnetic field inhibits RANKL-dependent osteoclastic differentiation in RAW264.7 cells through the Ca(2+)-calcineurin-NFATc1 signaling pathway. Biochem. Biophys. Res. Commun. 482 (2), 289–295. 10.1016/j.bbrc.2016.11.056 27856256

[B38] Zhang L. Mingtao D. Dai P. Chen W. Fang N. Chen L. (2013). Eucommia leaf promotes rat osteoblast proliferation by activating the phosphorylation of ERK and AKT. Chin. J. Osteoporos. 19, 217–220.

[B39] Zhao X. Wang Y. Nie Z. Han L. Zhong X. Yan X. (2020). Eucommia ulmoides leaf extract alters gut microbiota composition, enhances short-chain fatty acids production, and ameliorates osteoporosis in the senescence-accelerated mouse P6 (SAMP6) model. Food Sci. Nutr. 8 (9), 4897–4906. 10.1002/fsn3.1779 32994951 PMC7500782

