# Supplementary material for: Anti-osteoporotic effects of Yi Mai Jian on bone metabolism of ovariectomized rats
Source: Front Pharmacol. 2024 Mar 28;15:1326415. doi: 10.3389/fphar.2024.1326415 (PMC11007778; doi:10.3389/fphar.2024.1326415)
Supplement: Supplementary file 1 [file DataSheet1.pdf]

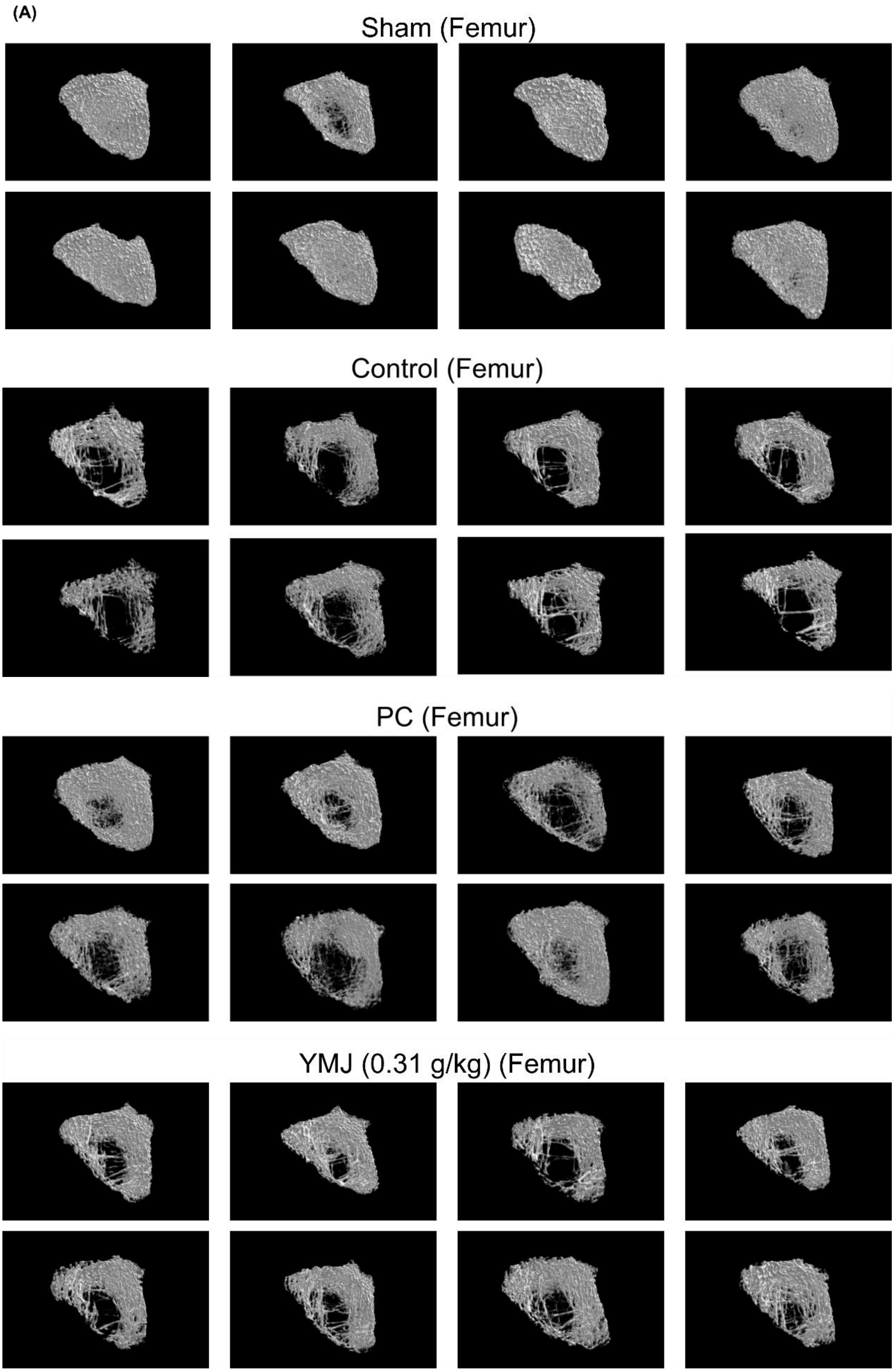
(A)
Sham (Femur)
Control (Femur)
PC (Femur)
YMJ (0.31 g/kg) (Femur)

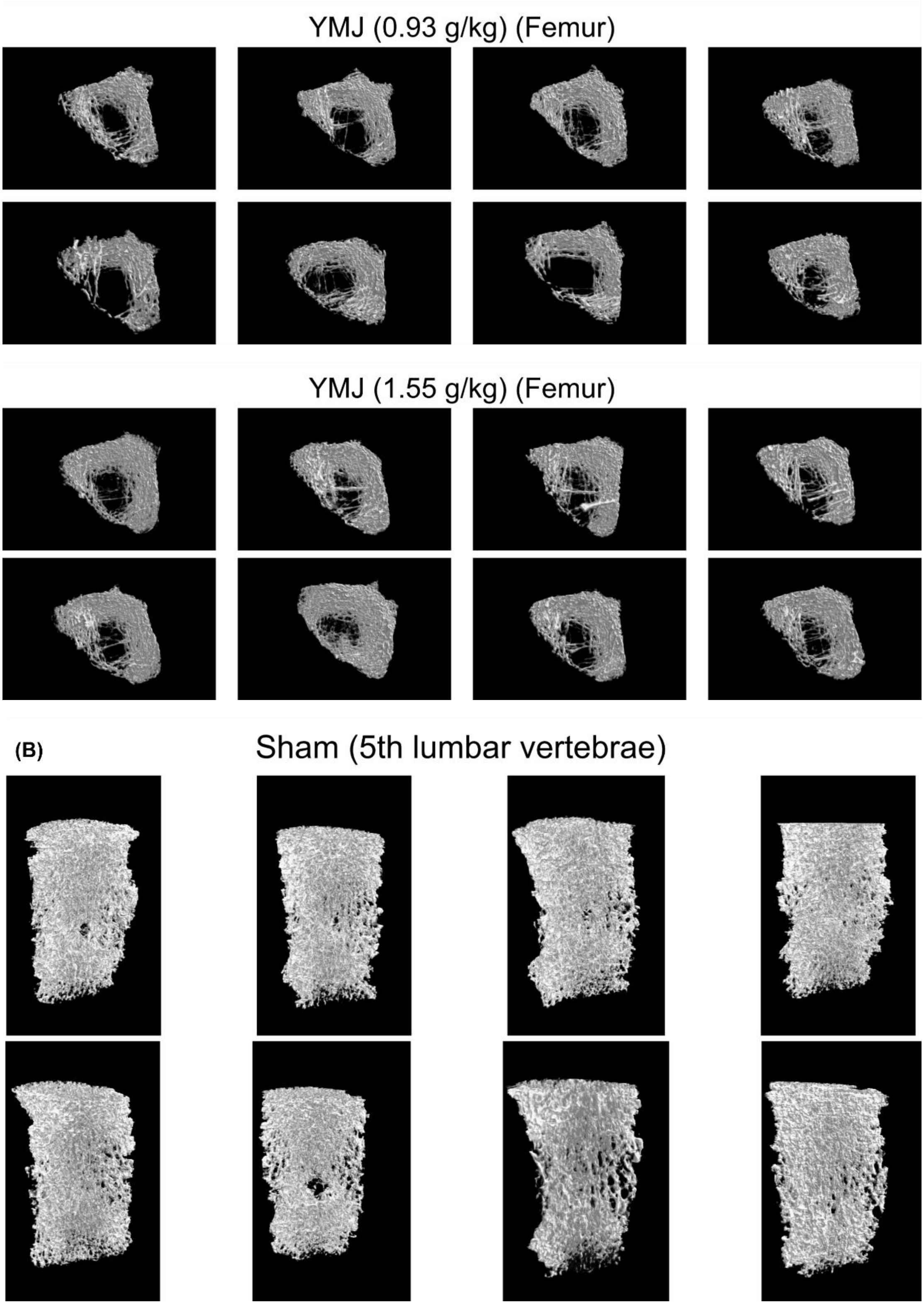
YMJ (0.93 g/kg) (Femur)
YMJ (1.55 g/kg) (Femur)
(B)
Sham (5th lumbar vertebrae)

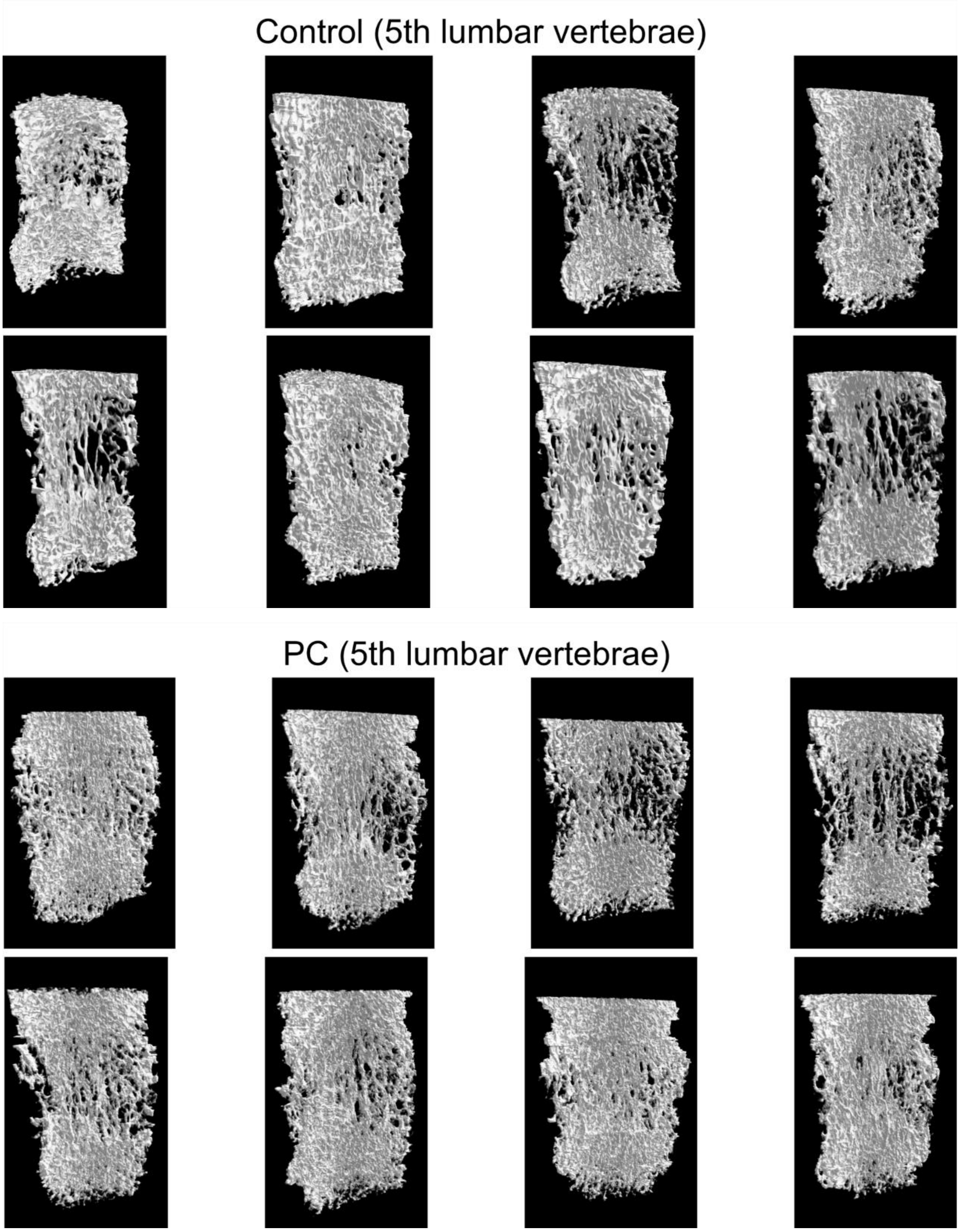
Control (5th lumbar vertebrae)
PC (5th lumbar vertebrae)

YMJ (0.31 g/kg) (5th lumbar vertebrae)

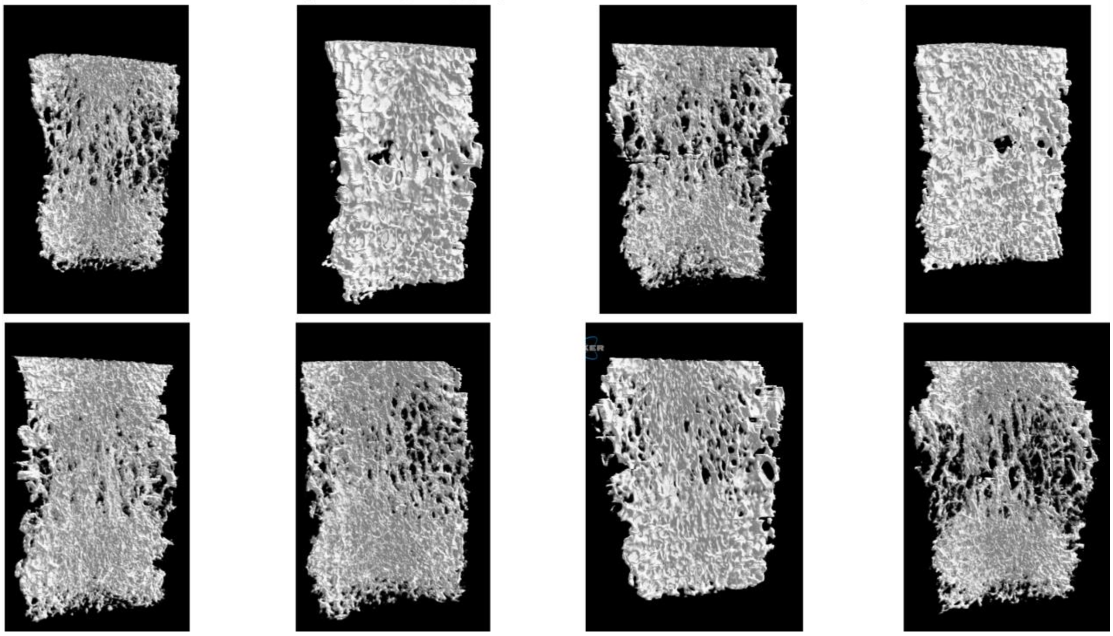

YMJ (0.93 g/kg) (5th lumbar vertebrae)

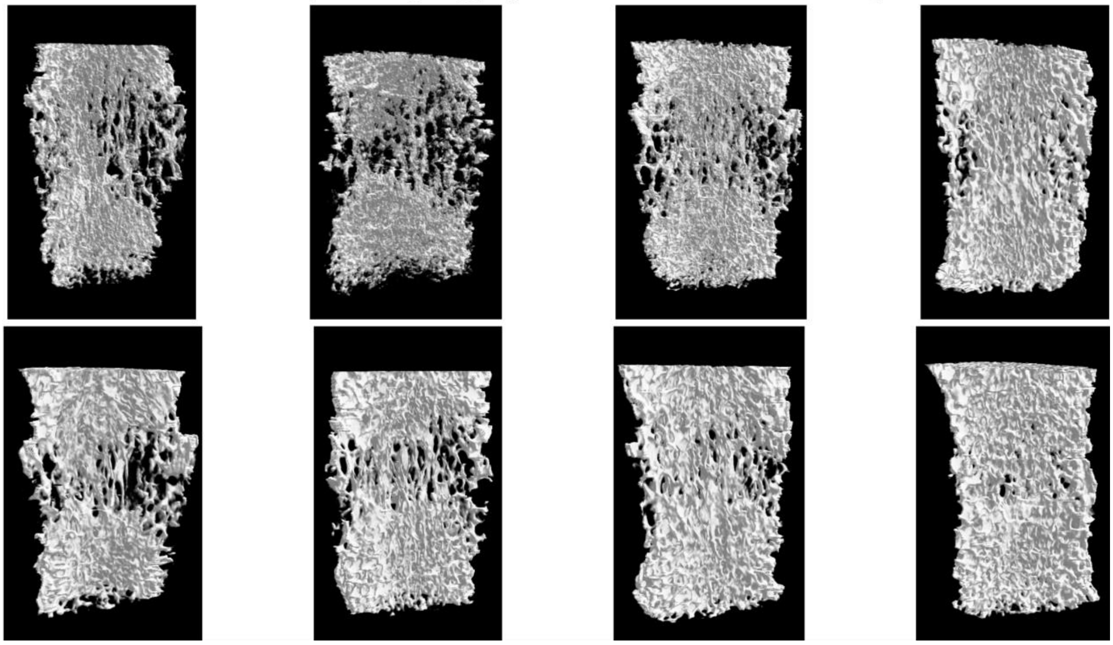

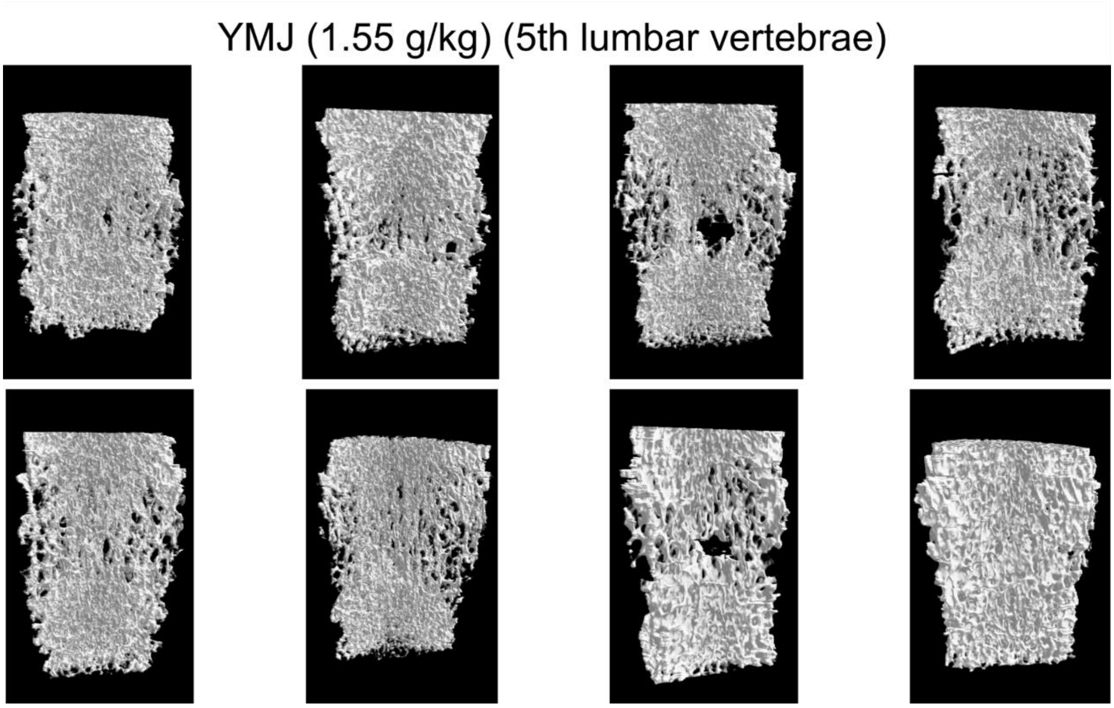

Supplemental Figure 1 Micro-computed tomography (micro-CT) images of the femur bone (A) and the 5th lumbar vertebrae (B) of 8 rats after YMJ treatment for 114 days.

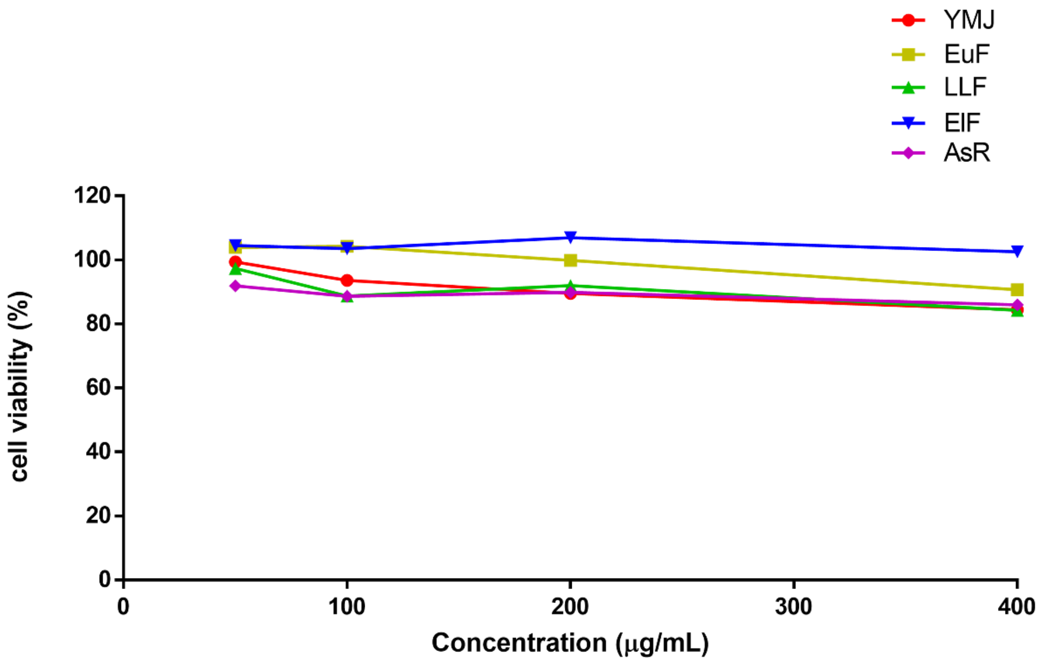

Supplemental Figure 2 The cell viability of YMJ and the extracts of its constituent herbs in NIH-3T3 cells.
